# Outcomes and Toxicity of Adult Medulloblastoma Treated with Pediatric Multimodal Protocols: A Single-Institution Experience

**DOI:** 10.32604/or.2025.067948

**Published:** 2025-11-27

**Authors:** Antonio Ruggiero, Dario Talloa, Alberto Romano, Giorgio Attinà, Stefano Mastrangelo, Palma Maurizi, Tommaso Verdolotti, Gianpiero Tamburrini, Silvia Chiesa, Rina di Bonaventura, Pier Paolo Mattogno, Alessandro Olivi, Alessio Albanese

**Affiliations:** 1 Pediatric Oncology Unit, Fondazione Policlinico Universitario Agostino Gemelli IRCCS, Rome, 00168, Italy; 2 Department of Women and Child Health and Public Health, Università Cattolica del Sacro Cuore, Rome, 00168, Italy; 3 ARC Advanced Radiology Center (ARC), Department of Oncological Radiotherapy and Hematology, Fondazione Policlinico Universitario Agostino Gemelli IRCCS, Rome, 00168, Italy; 4 Pediatric Neurosurgery, Fondazione Policlinico Universitario A. Gemelli, IRCCS, Rome, 00168, Italy; 5 Department of Neuroscience, Section of Neurosurgery, Università Cattolica del Sacro Cuore, Rome, 00168, Italy; 6 Gemelli Advanced Radiotherapy, Fondazione Policlinico Universitario A. Gemelli IRCCS, Rome, 00168, Italy; 7 Neurosurgery Unit, Department of Neurosciences, Fondazione Policlinico Universitario Agostino Gemelli IRCCS, Rome, 00168, Italy; 8 Neurosurgery Unit, Department of Neurosciences, Università Cattolica del Sacro Cuore, Rome, 00168, Italy

**Keywords:** Medulloblastoma, toxicity, adult, chemotherapy, children

## Abstract

**Background:**

Adult medulloblastoma (MB) represents less than 1% of central nervous system malignancies, lacking standardized therapeutic approaches due to its rarity. This retrospective single-center analysis aimed to assess survival outcomes and treatment-associated toxicities in adult MB patients managed with pediatric-derived protocols.

**Methods:**

Eighteen patients (≥18 years) with MB treated at Fondazione Policlinico Universitario Agostino Gemelli Istituto di Ricovero e Cura a Carattere Scientifico (IRCCS) (January 1997–January 2024) were analyzed. All received craniospinal radiotherapy with posterior fossa boost, followed by adjuvant chemotherapy utilizing pediatric regimens (PNET3, PNET4, PNET5, or high-risk protocols incorporating high-dose chemotherapy with autologous stem cell rescue). Primary outcomes included overall survival (OS) and progression-free survival (PFS). Secondary analyses focused on comprehensive toxicity assessment.

**Results:**

The cohort included 11 males and 7 females (median age: 23 years). Metastatic disease was present in 6 patients (33%) at diagnosis. Histopathological distribution showed classic MB (55.5%), desmoplastic/nodular (39%), and large cell/anaplastic variants (5.5%). Molecular subgrouping (available in 6 patients) identified SHH subgroup in four cases and WNT subgroup in two. Three-year and five-year overall survival rates reached 94.5% and 88.8%, respectively. Treatment-related adverse events included grade 3–4 hematologic toxicities, clinically significant weight loss, and grade ≥3 neurological and ototoxic complications. These toxicities necessitated treatment modifications including dose adjustments, cycle delays, and occasional early discontinuation.

**Conclusions:**

Adult MB patients treated with pediatric-adapted protocols demonstrated excellent long-term survival outcomes, comparable to or surpassing historical data. Despite frequent toxicity requiring treatment modifications, these regimens proved feasible with acceptable risk-benefit profiles. These results support implementing modified pediatric protocols for adult MB management. Future multicenter investigations with larger cohorts are essential for refining risk stratification, optimizing treatment intensity, and evaluating long-term outcomes in this rare malignancy.

## 1 Introduction

Medulloblastoma (MB), first characterized by Cushing and Bailey in 1925, is an aggressive grade IV embryonal tumor originating in the posterior cranial fossa [1]. MB shows a significant difference in frequency between age groups: it represents 15%–20% of all central nervous system (CNS) tumors in children aged 0–14 years, but occurs much less frequently in adults, accounting for less than 1% of CNS malignancies with an estimated incidence of only 0.6–1 case per million adults annually [2–4].

Treatment approaches for MB have evolved significantly since the 1950s, with particularly notable success in pediatric protocols. The integration of craniospinal radiotherapy with chemotherapy in the 1970s has revolutionized pediatric management, yielding current 5-year survival rates exceeding 80% for standard-risk cases and 60%–70% for high-risk pediatric patients [5,6]. Adult treatment has not consistently achieved comparable success rates, as patients frequently receive either modified pediatric regimens or adult-specific protocols that have not undergone the same rigorous multicenter validation process characteristic of pediatric trials. This disparity stems largely from biological differences in tumor behavior between age groups and the relative rarity of adult MB, which complicates the establishment of standardized treatment protocols [7].

Risk stratification in MB management has traditionally depended on a multifactorial assessment incorporating patient age at diagnosis, presence and extent of metastatic disease, degree of surgical resection achievable, and specific histological subtype characteristics, with these parameters serving as the cornerstone for treatment protocol selection and prognostic evaluation [8–10]. The Chang classification system (M0–M4) continues to guide metastatic staging despite its limitations in characterizing metastatic lesions beyond anatomical location. This classification creates particular challenges in risk assessment, as patients classified as M1 are now considered high-risk, despite ongoing debates regarding their prognosis compared to patients with solid metastases. The persistence of residual tumor exceeding 1.5 cm^2^ post-resection necessitates treatment intensification in established protocols, though recent clinical investigations challenge assumptions about subtotal resection inevitably yielding inferior outcomes compared to gross total resection, particularly when considering potential neurological morbidity associated with aggressive surgical approaches [9,10].

The identification of four molecular subgroups (WNT, SHH, Group 3, Group 4) defined by specific molecular alterations including pathway activations, chromosomal aberrations, and distinct mutational profiles has transformed MB classification with important age-related implications [5,11,12]. WNT tumors demonstrate excellent prognosis in children regardless of treatment intensity, while adult WNT tumors may exhibit less favorable outcomes [13]. SHH subgroup tumors predominate in both infants and adults but display markedly different genetic alterations and clinical behaviors between these age groups, necessitating different therapeutic approaches [14,15].

Adult MB demonstrates a different distribution of molecular subtypes compared to pediatric cases, with SHH subgroup tumors representing approximately 60% of adult cases vs. only 30% in children. Group 3 tumors, which carry the poorest prognosis in children, are exceedingly rare in adults, while Group 4 tumors occur in both populations but may exhibit different biological behavior across age groups [16].

Pediatric MB protocols have emerged from sequential multicenter trials, establishing well-defined regimens that balance efficacy against toxicity [17–19]. These typically incorporate risk-adapted craniospinal irradiation followed by intensive multiagent chemotherapy including cisplatin, vincristine, cyclophosphamide, and etoposide [20,21]. Children generally demonstrate superior tolerance to these intensive regimens with appropriate supportive care [21–23]. In contrast, adult MB treatment lacks standardized protocols specifically designed for adult physiology and tumor biology. Many treatment centers apply modified pediatric regimens to adult patients, but these adaptations often result in increased toxicity leading to treatment interruptions, dose reductions, or premature termination of therapy. Hematological toxicity, neurotoxicity, and ototoxicity represent particularly challenging adverse effects in the adult population [24–26].

The integration of molecular profiling into clinical decision-making offers promising avenues for improving MB management across age groups. For pediatric patients, this approach has already enabled treatment de-escalation for favorable-risk groups (particularly WNT tumors) while maintaining excellent survival outcomes [11,14]. Similar molecular-guided treatment customization may benefit adult patients, though adult-specific prognostic markers require further validation through larger cohort studies.

Our institution has pioneered an approach applying pediatric treatment protocols to adult MB patients, with particular emphasis on understanding the role of adjuvant chemotherapy at initial diagnosis and assessing treatment tolerance. This strategy aims to overcome the historical disparities in outcomes between pediatric and adult populations by leveraging the extensively validated pediatric protocols with appropriate modifications for adult physiology. Our experience suggests that with careful management of toxicities and individualized supportive care, many adult patients can successfully complete modified pediatric regimens with improved outcomes compared to traditional adult approaches. This work contributes to addressing the significant gap in evidence-based treatment guidelines for adult MB. It provides valuable insights into the feasibility and efficacy of applying pediatric protocols across age groups for this rare but aggressive malignancy.

## 2 Materials and Methods

### 2.1 Study Rationale

Management of MB in adult patients remains challenging due to the absence of universally accepted guidelines and conflicting evidence regarding treatment efficacy and tolerability. We performed a retrospective analysis of adult MB patients treated with pediatric protocols at our institution to evaluate long-term survival outcomes and comprehensively assess treatment-related toxicities, with particular focus on regimen adherence and completion rates.

### 2.2 Study Design and Patient Selection

This single-center retrospective study analyzed data from 18 consecutive adult patients with MB admitted to the Pediatric Oncology Unit, Fondazione Policlinico Universitario Agostino Gemelli IRCCS, between 01 January 1997 and 01 January 2024. The study was approved by the Institutional Review Board of Università Cattolica Sacro Cuore-Policlinico Gemelli IRCCS (number DIPUSVSP-21-03-252). All patients provided written informed consent prior to enrolment.

The inclusion criteria for this study require participants to be at least 18 years of age at the time of their MB diagnosis. All participants must have a histopathologically confirmed diagnosis of MB. Additionally, participants must have received treatment using pediatric chemotherapy protocols rather than adult treatment regimens. Complete documentation of toxicity data throughout both the treatment period and subsequent follow-up care must be available for each participant included in the study.

The exclusion criteria eliminate participants who were younger than 18 years at diagnosis from the study population. Patients without a confirmed histological diagnosis of MB were also excluded, as were those with incomplete documentation of treatment-related toxicities during either the active treatment phase or follow-up period. Finally, patients who received treatment with chemotherapy regimens designed for adult populations rather than pediatric protocols were excluded from participation in this research.

### 2.3 Study Objectives

The primary objective was to determine the overall survival (OS) and progression-free survival (PFS) at 3- and 5-year. Secondary objectives included descriptive analysis of short-term and long-term toxicities associated with adjuvant chemotherapy in adult MB patients and characterization of the clinical features of adult MB.

### 2.4 Data Collection

Medical records were analyzed to extract the following information: age at diagnosis; sex; histological diagnosis and molecular subtyping (when available); primary tumor location; date of diagnosis; presence of metastatic disease at diagnosis; neurosurgical procedure details and timing; chemotherapeutic agents administered; radiotherapy characteristics (timing, dosage, target volumes); treatment modifications (dose reductions, delays, discontinuations); hematological toxicities; audiological toxicities; neurological toxicities; nutritional difficulties and weight loss; disease recurrence; minimum 12-month follow-up.

### 2.5 Disease Classification and Risk Stratification

Post-operative magnetic resonance imaging (MRI) was performed within 48 h of surgery and interpreted by a neuroradiologist to determine the extent of resection. When MRI was unavailable, the extent of resection was determined from the surgeon’s operative report. Patients in this study were categorized into distinct risk groups based on specific clinical and radiological characteristics. The standard-risk group included patients who showed no evidence of metastatic disease when evaluated through MRI or cerebrospinal fluid (CSF) analysis, and who had residual tumor measuring less than 1.5 cm^2^ following initial treatment. In contrast, patients were classified as high-risk if they demonstrated the presence of metastatic disease, defined as Chang stage M1 or higher, or if their residual tumor exceeded 1.5 cm^2^ in size. Molecular subtyping was performed when feasible using a combination of immunohistochemistry, DNA methylation arrays, next-generation sequencing, and targeted reverse transcription polymerase chain reaction (RT-PCR) for mRNA expression analysis.

### 2.6 Treatment Protocols

All patients received craniospinal radiation therapy (RT) with a boost to the primary MB site. The total radiation dose varied according to treatment protocol. Patients received adjuvant chemotherapy with different pediatric regimens, including PNET3 (vincristine, etoposide, carboplatin, and cyclophosphamide), PNET4 (vincristine, cisplatin, and lomustine, and), PNET5 (vincristine, cisplatin, lomustine, and cyclophosphamide), and other combined regimens.

For patients treated with the PNET3 protocol, chemotherapy was administered after the completion of radiotherapy (35 Gy to the craniospinal axis with a boost to 54–58 Gy on the posterior cranial fossa), rather than before RT as specified in the original protocol. This modification was based on evidence from the German HIT91 study indicating that pre-RT chemotherapy was associated with myelotoxicity leading to RT delays or interruptions, and findings from the SIOPII trial demonstrating no benefit from pre-RT chemotherapy [17,18,20].

### 2.7 Toxicity Assessment

Treatment-related toxicities were classified according to the Common Terminology Criteria for Adverse Events v5.0 (CTCAE) of the National Cancer Institute (NCI) (published 27 November 2017), with the exception of audiological toxicities, which were evaluated using the Brock criteria based on tonal audiometry assessment. Data collection focused on: (a) toxicities commonly observed in adults with MB treated with pediatric protocols: platinum-induced hearing loss; chemotherapy-associated neurological toxicity; nutritional difficulties and weight loss; (b) hematological toxicities frequently associated with treatment delays, modifications, or discontinuations

### 2.8 Statistical Analysis

Continuous variables were expressed as median and standard deviation (SD). Categorical variables were presented as absolute frequencies and percentages and were compared using the Chi-squared test. Survival analyses were performed using the Kaplan-Meier method and compared with the log-rank test. XLSTAT software (ver. 2021.3.1, Addinsoft, Paris, France) was used for statistical analysis.

Overall survival (OS) is defined as the time from the start of treatment to the time of death from any cause and was censored at the date of last follow-up. Progression-free survival (PFS) is defined as the time from the start of treatment to either death, progression of disease, or development of a secondary tumor and was censored at the time of last follow-up.

## 3 Results

### 3.1 Patient Characteristics

During the study period, 18 adult patients with a presumptive diagnosis of MB were initially evaluated. One patient was excluded following histopathological re-evaluation, which revised the diagnosis to pineoblastoma. The final cohort consisted of 18 patients treated at our center between 01 January 1997 and 01 January 2024.

The cohort comprised 11 males (61%) and 7 females (39%), with a mean age at diagnosis of 25.5 years (SD 6.66) and a median age of 23 years. Disease staging was performed according to the Chang classification, with all patients undergoing pre-operative MRI, post-operative MRI within 48 h, and lumbar puncture for CSF cytological analysis [8].

Six patients (33%) presented with metastatic disease at diagnosis (2 were Chang M1 stage and 4 M3 stage), while 12 patients (67%) had localized disease confined to the posterior cranial fossa. All non-metastatic patients had post-surgical residual tumor absent or <1.5 cm^2^. No patients presented with or developed M4 disease during the course of illness or follow-up.

Histological analysis revealed classic MB in 10 patients (55.5%), desmoplastic/nodular MB (MB-DN) in 7 patients (39%), and large cell/anaplastic MB (LCA) in 1 patient (5.5%). No cases of MB with extensive nodularity (MB-MBEN) were observed. Molecular characterization was available for 6 patients (33.3%), with 4 classified as the SHH subgroup and 2 as the WNT subgroup, further limiting molecular subgroup analysis. No patients had comorbidities that would have contraindicated chemotherapy at diagnosis.

### 3.2 Treatment Characteristics

Among the 12 standard-risk patients, 6 (33%) received treatment according to the PNET3 protocol, 4 (22%) according to PNET4, and 2 (11%) according to PNET5. The evolution in treatment protocols reflected changes in institutional practice over the study period, with patients receiving the standard protocol that was active at the time of their diagnosis. Of the 6 high-risk patients, 4 (22%) received tandem thiotepa high-dose chemotherapy (HDCT) followed by autologous stem cell transplantation (ASCT), and 2 (11%) received alternative high-risk MB chemotherapy regimens including thiotepa and carboplatin.

All patients received craniospinal radiotherapy with a boost to the posterior cranial fossa. The radiation dose varied according to risk classification and treatment protocol (23.4–36 Gy craniospinal irradiation, SD 8.9; 54–58 Gy posterior cranial fossa boost, SD 1.4). Radiotherapy was initiated within 40 days of surgery for all patients and completed within 50 days of initiation. Three patients (17%) required temporary interruption of radiotherapy due to hematological toxicities (neutropenia <500/mm^3^, thrombocytopenia <20,000/mm^3^).

All six high-risk patients received HDCT/ASCT with thiotepa-based conditioning regimens, although at different timepoints in their treatment course. Four patients received HDCT/ASCT as consolidation therapy, while two received mobilizing chemotherapy with carboplatin/etoposide followed by autologous transplantation with thiotepa conditioning as salvage therapy after disease relapse.

During treatment, patients received transfusion support according to institutional protocols: packed red blood cell transfusions for hemoglobin <7 g/dL, platelet transfusions for platelet counts <20,000/mm^3^, and granulocyte colony-stimulating factor for persistent moderate/severe neutropenia or neutropenia requiring treatment interruption (neutrophil count <500/mm^3^ for >2 weeks).

### 3.3 Survival Outcomes

Patients were followed for a median of 42 (range 12–264) months. All patients (n = 18) were alive at the 12-month follow-up post-diagnosis. At this time point, 17 patients (94.4%) exhibited disease remission, while one patient (5.6%) demonstrated disease progression at 9 months post-diagnosis. This non-responsive patient (with anaplastic features, and M3 stage) failed to respond to subsequent therapeutic interventions and succumbed to the disease at 35 months post-diagnosis.

One patient developed disease recurrence 34 months after initial diagnosis (20 months following the achievement of complete remission) and died 52 months from the time of original diagnosis. Another patient manifested multiple disease relapses and survived for 116 months after initial diagnosis before mortality occurred.

**Figure 1:**
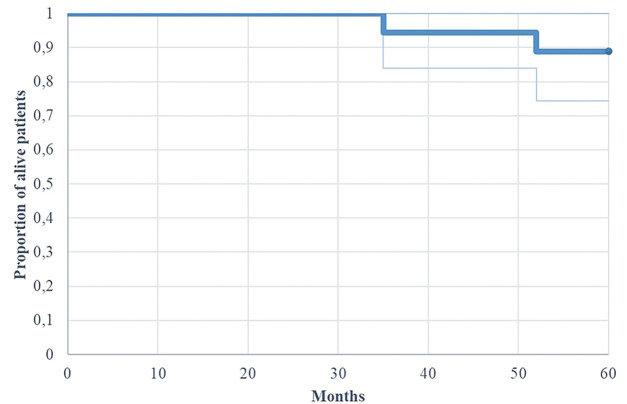
Kaplan-Meier of overall survival (OS) for patients with adult MB (n = 18)

OS at 3 years and 5 years was 94.5% and 88.8%, respectively, for the entire cohort (Fig. 1 shows the Kaplan-Meier OS curve for the entire cohort). When stratified by risk classification, standard-risk patients demonstrated 3-year and 5-year OS rates of 100% and 91.6%, respectively, while high-risk patients exhibited rates of 83.3%, both at 3 and 5 years (*p* = 0.92 for 5-year OS comparison).

**Figure 2:**
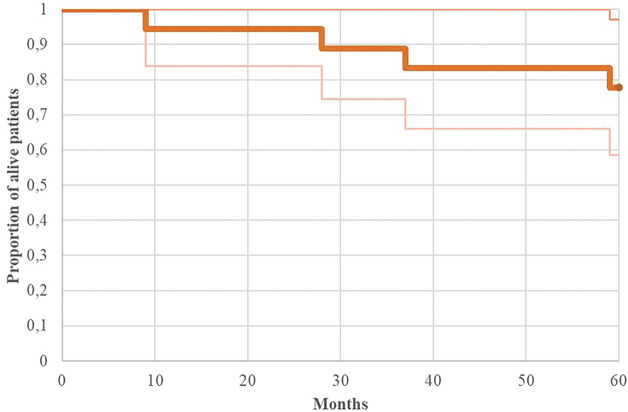
Kaplan-Meier of progression-free survival (PFS) for patients with adult MB (n = 18)

Regarding PFS, the entire cohort demonstrated 3-year and 5-year rates of 83.3% and 77.8%, respectively (Fig. 2 illustrates the Kaplan-Meier progression-free survival curve for all patients). Risk stratification revealed 3-year and 5-year PFS rates of 91.6% for standard-risk patients, compared to 66.6% and 50% for high-risk patients, respectively (*p* = 0.71 for 5-year PFS comparison).

### 3.4 Treatment-Related Toxicities

Among the observed adverse events, grade ≥3 hematological toxicities were predominant, occurring in 11 patients (61.1%). Chemotherapy-induced neurological complications were documented in 5 patients (27.7%), primarily manifesting as sensory-motor and autonomic neuropathies. Clinical presentations included decreased lower extremity strength, limb paresthesias, and constipation. Vincristine administration was identified as the principal causative agent, necessitating dose modifications in the majority of affected cases.

Severe ototoxicity (grade ≥3, characterized by hearing threshold exceeding 40 dB at frequencies of 4000 Hz and above) was observed in 4 patients (22.2%). Nutritional status deterioration was notable, with 13 patients (72.2%) exhibiting weight reduction exceeding 10% during the treatment course (Table 1 presents the detailed distribution of treatment-related adverse events by grade and frequency). This nutritional compromise was evident in both metastatic high-risk patients and those presenting with poor baseline performance status.

**Table 1:** Frequency of treatment-related toxicities (Grade 3–4) stratified by risk classification

Adverse event	All patients (N = 18) (%)	High-risk patients (N = 6)	Standard-risk patients (N = 12)	*p* value
Hematological toxicities	11 (61.1)	6 (100.0)	5 (41.6)	0.06
Weight loss	13 (72.2)	5 (83.3)	8 (66.6)	0.45
Chemotherapy-induced neurotoxicity	5 (27.7)	1 (16.6)	4 (33.3)	0.45
Ototoxicity	4 (22.2)	0 (0.0)	4 (33.3)	0.31

Note: Toxicity was graded according to the Common Terminology Criteria for Adverse Events (CTCAE) version 5.0.

Treatment-related adverse events considerably influenced therapeutic protocol adherence. Chemotherapy cycle initiation delays were observed in 7 patients (38.8%), while dosage modifications of at least one chemotherapy agent were necessary in 10 patients (55.6%). Premature discontinuation or temporary suspension of chemotherapeutic intervention occurred in 8 patients (44.4%) due to treatment-emergent toxicities.

## 4 Discussion

The survival outcomes observed in adult MB patients treated at our institution with pediatric-inspired chemotherapy protocols are encouraging and align with previously reported results for adult cohorts receiving combined chemoradiotherapy regimens [27–29]. Our patients achieved a 3-year OS of 94.5% and a 5-year OS of 88.8%, outcomes that compare favorably with the 5-year OS of 78% reported by Chen et al. (2022) for adults treated with chemoradiotherapy and with the 66% OS seen in patients receiving radiotherapy alone [30]. Similarly, Kocakaya et al. (2016) documented a 71% 9-year survival in comparable populations [31].

Contrary to the findings by Ma et al. (2020), who identified age as an independent prognostic factor (Hazard Ratio (HR) 1.01–1.02, *p* < 0.001), our data did not demonstrate a statistically significant association between age and survival (HR 0.824–1.220, *p* = 0.978) [7]. Moreover, treatment timing across our cohort remained within clinically acceptable limits, with no delays exceeding 30 days from surgery to chemotherapy initiation or 50 days from radiotherapy completion to subsequent chemotherapy commencement.

A major limitation of our study pertains to the availability of molecular profiling, which was completed in only six patients (four MB-SHH and two MB-WNT). This highlights an urgent need to implement routine molecular classification at diagnosis, in alignment with current World Health Organization (WHO) recommendations, to enhance risk stratification and guide treatment personalization in adult MB. However, although molecular subgrouping was available in only six patients, the prognostic implications of these classifications deserve consideration. WNT subgroup tumors are associated with the most favorable prognosis, with survival rates exceeding 90% in pediatric cohorts, while SHH tumors exhibit intermediate outcomes with constitutive activation of the SHH pathway through mutations in GLI1, GLI2, SUFU, and PTCH1 genes [32,33]. The two WNT patients in our series both achieved long-term survival, consistent with literature reports of excellent outcomes in this subgroup. Conversely, among the four SHH patients, survival outcomes varied, reflecting the known heterogeneity within this molecular classification. The lack of Group 3 and Group 4 patients in our molecularly characterized cohort limits comparative analysis, though Group 3 tumors typically demonstrate the worst survival outcomes with rates under 60% at 5 years. These findings underscore the critical importance of implementing routine molecular profiling at diagnosis to guide risk-adapted therapeutic strategies, particularly given the potential for treatment de-escalation in WNT patients and targeted approaches for SHH tumors.

While statistical comparisons with historical pediatric cohorts are constrained by our limited sample size, it is noteworthy that among six high-risk patients in our series, four who underwent tandem high-dose chemotherapy with autologous stem cell rescue were alive at last follow-up (mean follow-up: ~4 years) [32]. Although maintenance therapy could not be completed in three patients due to persistent hematologic toxicity, these findings are consistent with outcomes in pediatric populations, where 5-year OS exceeds 80% in standard-risk and 60–70% in high-risk subgroups [33]. These preliminary results suggest that adult patients may benefit from intensified regimens analogous to those used in pediatric protocols.

Toxicity remains a significant concern when translating pediatric regimens to adult patients. Hematologic toxicity was frequent and often severe. Craniospinal irradiation is known to impair bone marrow function, contributing to treatment delays or dose modifications that may ultimately affect disease control [16,34–36]. In our cohort, grade ≥3 neutropenia, thrombocytopenia, and/or anemia occurred in 61.1% of patients. This burden of toxicity led to chemotherapy discontinuation in 44.4%, dose delays in 38.8%, and dose reductions in 55.6% of cases. Although hematologic events were the primary driver of these modifications, the role of neurologic complications and nutritional decline warrants further investigation, particularly in assessing their potential impact on adherence and survival.

Audiologic toxicity, specifically cisplatin-induced hearing loss, was also notable. All patients who developed hearing impairment had received cisplatin-based regimens. In one case, early-onset ototoxicity prompted substitution of cisplatin with carboplatin, an agent with established efficacy and a more favorable auditory toxicity profile in MB [37–39]. These findings emphasize the necessity of routine audiometric monitoring during treatment to facilitate timely modifications. The multifactorial etiology of hearing loss—encompassing surgical trauma and cranial irradiation—also underscores the relevance of emerging strategies such as proton therapy, which may reduce ototoxic risk.

Neurologic toxicities, particularly vincristine-induced peripheral neuropathy, affected 27.7% of patients. Though generally reversible with dose adjustments or following treatment cessation, such toxicities may transiently impair functional status and quality of life. Vincristine-associated dysautonomia further contributed to gastrointestinal symptoms, including constipation, exacerbating nutritional challenges during therapy. Management of neurological toxicities was primarily reactive, with vincristine dose reductions or temporary discontinuation implemented upon symptom onset. Systematic monitoring included regular neurological assessments, though formal neuropathy grading scales were not consistently applied throughout the study period.

Nutritional compromise was prevalent, with 72.2% of patients experiencing >10% weight loss during chemoradiotherapy. This multifactorial complication—driven by mucositis-induced dysphagia, gastrointestinal toxicity, and overall treatment burden—significantly diminished patient well-being and autonomy. While all patients received symptomatic management and prophylactic antiemetics, enteral nutrition (via nasogastric or gastrostomy tubes) and total parenteral nutrition (TPN) were required in select high-risk patients, particularly during periods of severe mucositis or disease progression. While these measures were predominantly reactive rather than prophylactic, they proved effective in managing treatment-related complications and maintaining therapy feasibility.

The cumulative burden of toxicity translated into substantial treatment modification, highlighting the need for adult-specific dose-intensity optimization strategies. These modifications, while necessary for tolerability, may carry implications for treatment efficacy and long-term outcomes.

Due to the inherent rarity of adult MB and the resultant small sample size, this study is limited in its ability to draw definitive conclusions regarding prognostic factors or regimen-specific outcomes. The limited sample size of our cohort (n = 18) inherently restricts the generalizability of our findings and precludes robust statistical analysis of prognostic factors, particularly in subgroup comparisons between risk categories and molecular subtypes, which is a common limitation in studies of rare malignancies such as adult MB.

Nevertheless, our findings underscore the feasibility and potential efficacy of pediatric-inspired multimodal therapy in this population, while also illustrating the challenges posed by treatment-related toxicities. Collaborative multicenter studies with larger cohorts and systematic molecular profiling are essential to refine risk-adapted treatment strategies and improve outcomes for adults with MB.

## 5 Conclusions

The results obtained confirm that the use of adjuvant chemotherapy with schemes derived from pediatric protocols in adult patients with MB leads to a survival rate comparable to, and in some cases, even in the absence of statistical comparison, superior to available historical. Haematological, neurological, audiological toxicities, together with the nutritional difficulties observed, were relevant and led to interruptions, delays and reductions of chemotherapy dose in a significant percentage of patients. Nevertheless, the majority of the patients in study completed the treatment, and no death related to treatment or severe long-term sequelae were observed, indicating an overall acceptable survival and tolerability. Prospective studies evaluating the impact of more recent treatment protocols in terms of survival and long-term toxicities are necessary in order to identify the appropriate chemotherapy timing for different risk classes and the potential superiority of specific therapy regimens.

Adult MB still constitutes a field of exploration due to its rarity and due to its biological differences with pediatric MB. The rarity of the diseas determines several issues: some directly impact the single patient such as delay in diagnosis, receiving treatment within appropriate time frames, difficulties in finding specialists and referral centers specialized in the management of MB in adults; other issues impact indirectly on the prognosis of the adult patient with MB by hindering research, such as the difficulty in conducting clinical studies due to small sample sizes and the low interest of pharmaceutical companies in developing innovative therapeutic agents targeted for adults.

Being a “borderline” disease in terms of onset age and clinical characteristics, it is considered necessary for these patients to be treated by a multidisciplinary team that should include a neurosurgeon (with expertise in PCF and MB surgery), a pathologist (with possibility of centralized review and molecular analysis of the samples), a neuroradiologist, a pediatric oncologist (for the appropriate application and modulation of the chemotherapy regimen), a radiotherapist, a neurologist, a psychologist/therapist, and a palliative care specialized team. Collaboration between specialists is one essential criteria for the inclusion in the centre of the European Reference Network for the treatment of rare diseases (ERN, European Reference Network for rare diseases). The inclusion in an international reference network responsible for rare diseases is therefore a necessity for the management of adult MB patients, with predictable advantages for the patient in terms of faster access to experimental therapies, better management of chemo-radiotherapy in accordance with international protocols, resulting in benefits both from a medical, a social and a psychological perspective.

## Data Availability

The data that support the findings of this study are available from the Corresponding Author, [Antonio Ruggiero], upon reasonable request.
